# Special Issue “New Advances in Breast Imaging”

**DOI:** 10.3390/tomography8040142

**Published:** 2022-06-28

**Authors:** Daniele Ugo Tari

**Affiliations:** Department of Diagnostic Senology, District 12, Caserta Local Health Authority, Caserta LHA, 81100 Caserta, Italy; daniele.tari@aslcaserta.it

Breast cancer (BC) is the most common cancer in women of all ages, with more than 2 million diagnoses every year and a high economic and psychological impact on both the health care system and the population [1,2]. Additionally, male breast cancer should be considered, because even though it represents less than 1% of all male malignancies, its incidence has increased by 20–25% in the past few decades [3].

Breast imaging is one of the most exciting subspecialties in radiology. The importance of early diagnosis is widely confirmed, and the modalities of breast cancer screening are highly debated, with several guidelines proposed in the world [4,5].

In these challenging times, a well-established diagnostic–therapeutic care pathway (DTCP) is fundamental to guarantee an essential level of care since the COVID-19 pandemic has caused permanent changes to the practice of medicine [6,7].

Currently, the need for personalized medicine has also changed our vision of the field, giving even more importance to multimodality and to the integration of both artificial intelligence and human resources. Consequently, evaluating the application of radiomics could be of particular interest. Increased mammographic breast density is a well-established risk factor for the development of breast cancer, regardless of age or ethnic background [8]. The current gold standard for categorizing breast density consists of a radiologist estimation according to the American College of Radiology (ACR) Breast Imaging Reporting and Data System (BI-RADS) criteria [9]. It could be useful to establish the effect of the automated evaluation of breast density with the currently available software. In addition, evaluating how breast cancers are depicted by artificial intelligence-based computer-assisted diagnosis (AI-CAD) could also be of relevant interest.

Furthermore, examining the real impact of the most recent advancements in standard imaging techniques on breast cancer prevention, such as digital breast tomosynthesis (DBT) and automated breast ultrasound (ABUS), could be incredibly challenging, especially in women with dense breasts. In addition, contrast-enhanced spectral mammography (CESM) could be considered a true alternative to the most expensive magnetic resonance imaging (MRI) [10,11]. On the other hand, the evaluation of fast MRI could have a great impact on breast cancer prevention, especially in women at high risk [12].

This Special Issue aims to present and discuss the most recent advancements in breast imaging in order to strengthen the concept that breast imaging is a specific area that requires specific training and that a multimodality approach is the solution to guarantee a complete diagnosis for patients.

Original research articles and reviews are welcome. Research areas include (but are not limited to) all the aforementioned topics. Articles on breast cancer screening programs are welcome, especially those dealing with DBT and ABUS.

Finally, articles on breast interventional radiology, with particular regard to vacuum-assisted biopsy or excision systems, are also welcome.
